# DSM-5 and ADHD – an interview with Eric Taylor

**DOI:** 10.1186/1741-7015-11-204

**Published:** 2013-09-12

**Authors:** Eric Taylor

**Affiliations:** 1 Institute of Psychiatry, King’s College London, De Crespigny Park, London SE5 8AF, United Kingdom

## Abstract

In this podcast we talk to Prof Eric Taylor about the changes to the diagnosis of Attention Deficit Hyperactivity Disorder (ADHD) in DSM-5 and how these changes will affect clinical practice.

The podcast for this interview is available at: http://www.biomedcentral.com/sites/2999/download/Taylor.mp3.

## Introduction

**Figure 1:**
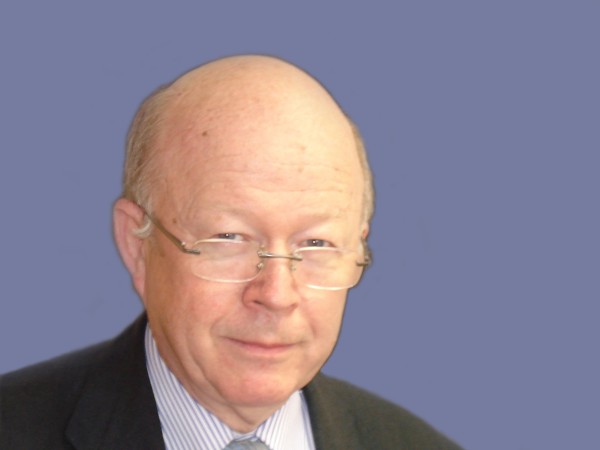
Eric Taylor.

Prof Eric Taylor is Emeritus Professor of Child and Adolescent Psychiatry at King’s College London, Institute of Psychiatry and is an honorary consultant at the Maudsley hospital. He has developed specialist clinics for child neuropsychiatry and higher training for child and adolescent psychiatry. His research has included longitudinal epidemiology, nosological distinctions within the ADHD spectrum, neuropsychology and neuroimaging, molecular genetics and treatment trials. Prof Taylor has chaired the NICE guidelines development group for ADHD, was senior author for the various European Guidelines from EUNETHYDIS and also a Trustee of the National Academy of Parenting Practitioners and a Non-Executive Director of the South London and Maudsley NHS Foundation Trust. He is a Fellow of the Academy of Medical Sciences, an Honorary Fellow of the Royal College of Psychiatrists, and a Board Member of the Association for Child and Adolescent Mental Health and Place2Be (providing mental health services to schools). Prof Taylor’s publications include more than 200 empirical scientific papers and several reviews, chapters, editorials and several books (Figure 1).

The podcast for this interview is available at: http://www.biomedcentral.com/sites/2999/download/Taylor.mp3.

## Edited transcript

### 1. What are the changes to the diagnosis of attention deficit hyperactivity disorder (ADHD) based on the fifth Edition of the Diagnostic and Statistical Manual of Mental Disorders (DSM-5)?

(i) ADHD is now classified as one of the neurodevelopmental disorders rather than being included as a disruptive disorder.

(ii) The diagnostic criteria that apply to adults have been relaxed and this reflects growing evidence that some people are still impaired by ADHD symptoms even if the symptoms have reduced in number. The criteria for children remain at six out of nine from a list of inattentiveness symptoms and/or six out of nine from a list of symptoms of hyperactivity and impulsiveness. Adults over 18, however, need only to display five out of the same lists. Furthermore, for adults being diagnosed for the first time, the criteria for onset in childhood have also been relaxed. Previously there had to have been “some impairment” before the age of seven. Now it is only necessary to show that symptoms were present (not impairment) before the age of twelve.

(iii) The traditional subtypes of predominantly inattentive, predominantly impulsive hyperactive and combined have been downgraded from subtypes to presentations. This reflects new knowledge that one subtype frequently changes into another.

(iv) Autism is no longer an exclusion and both autism and ADHD can be diagnosed together.

(v) Rapid and labile mood changes, which often accompany ADHD, now have a new diagnosis of their own. This is termed “Disruptive mood dysregulation disorder” (DMDD), which describes a group of children with frequent and severe outbursts of temper whose mood between tempers is angry or miserable.

### 2. What will be the impact of DSM-5 on the diagnosis of ADHD?

The impact is not likely to be great. Practitioners in the UK do not usually add up the symptoms in the way that DSM envisages. Instead, they match the case to their prototype idea of the disorder, as described by the 10^th^ version of the International Classification of Diseases (ICD-10) and in professional guidelines. The fundamental conceptualisation of the disorder has not been changed. There may be a modest increase in the recognition of ADHD in adult life, but this is happening anyway without the additional spur of DSM changes.

### 3. What are your views on these changes?

They are mostly tweaks to the diagnostic description and most of the changes are welcome. Some opportunities have been missed and these include the possibilities of moving to recognise the distinctive problems of attention deficit in the total absence of hyperactivity, the opportunity to move to a more dimensional system of describing psychopathology, and the notion of providing a clear system to describe severity, staging and impairment. The specific changes include:

(i) While ADHD has many features in common with disruptive disorders, I believe that the shift to “neurodevelopmental” is correct and timely. Like other neurodevelopmental problems, ADHD has an early onset, steady course, high male/female ratio, strong genetic influences and multiple associations in altered brain function.

(ii) I think it is correct to have made the adult diagnosis a little easier to achieve. User groups complain strongly that the NHS fails to recognise their difficulties even when they are presented to general psychiatry.

(iii) I agree that research has not been kind to the idea that there are valid subtypes. However, some of this failure of validity has to do with poor operational definitions. The “predominantly inattentive” subtype can apply to children who have as many as five of the hyperactivity symptoms, which is quite a lot, but fall just under the cut-off point. Nevertheless, inattentive children with no hyperactivity at all can still be seriously impaired but in a different way. Their school work and occupational success suffer from a wide range of neuropsychological problems. These problems deserve scientific study, but have not received this partly because they are not included in the existing diagnostic schemes.

(iv) Autism used to be excluded, but autism and ADHD frequently coexist. It is therefore important to recognise both disorders as patients with ADHD are treated successfully even when they have autism spectrum disorders.

(v) The new diagnosis of DMDD does not really have a full scientific basis. It has been included largely to tame an epidemic in the USA in which irritable children are diagnosed as having bipolar disorder, with a consequent massive increase in the use of powerful neuroleptic drugs even in the under-fives. I am inclined to doubt whether the existence of a new diagnosis will in fact reduce the epidemic of diagnosis, but luckily it has not reached the UK anyway.

### 4. How do you think these changes will affect clinical practice?

As indicated above, I do not think that there will be a big impact because of the different bases of practice in the USA and in the UK. There is, in any case, a continuing increase in the recognition and treatment of ADHD, and concomitantly in the prescription of stimulant medication. This change represents more accurate recognition, especially in adult life and even after the increase the UK is close to the bottom of the European tables in diagnosis and treatment. I hope that we will not catch up with the USA where ADHD is the commonest childhood diagnosis (around 5%) and stimulant medication is even more common (around 7%). I do not see anything in the changes that are likely to take us in that direction. The new condition of DMDD may, in practice, be hard to distinguish from the states of agitation and angry depression that often coincide with severe conduct problems in young people. Nevertheless, irritability is a very common reason for referral to Child and Adolescent Mental Health Services (CAMHS), and it is likely to be helpful if there is now a language within which to think about such presentations.

### 5. What in your opinion are the future directions for ADHD diagnosis?

It seems likely that advances in understanding the neurophysiological and neuropsychological alterations that are seen in ADHD will lead us towards a more discriminating recognition of conditions. If we can shift to formulations of the brain mechanisms involved, this could lead us to diagnoses that predict more closely to treatment response more closely and suggest new treatments for components of the disorder.

### 6. Where can I find out more?

See references [1-6].

## Competing interests

ET has no competing financial interests to declare but was a member of work groups for the revision of DSM.
